# The impacts of the scope of benefits expansion on hospice care among adult decedents: a nationwide longitudinal observational study

**DOI:** 10.1186/s12904-023-01146-z

**Published:** 2023-03-29

**Authors:** Tsung-Hsien Yu, Frank Leigh Lu, Chung-Jen Wei, Wei-Wen Wu

**Affiliations:** 1 Department of Health Care Management, National Taipei University of Nursing and Health Sciences, Taipei, Taiwan; 2 Department of Pediatrics, National Taiwan University Children’s Hospital, Taipei, Taiwan; 3 School of Medicine, National Taiwan University, No.1 Jen-Ai Road section 1 Taipei 100, Taipei, Taiwan; 4 Department of Public Health, Fu-Jen Catholic University, New Taipei, Taiwan; 5 School of Nursing, College of Medicine, National Taiwan University, Taipei, Taiwan; 6 Department of Nursing, National Taiwan University Hospital, Taipei, Taiwan

**Keywords:** Hospice care, Health Care utilization, Population characteristics

## Abstract

**Objectives:**

Compared to aggressive treatment for patients at the end stage of life, hospice care might be more likely to satisfy such patients’ need and benefits and improve their dignity and quality of life. Whether the reimbursement policy expansion affect the use of hospice care among various demographics characteristics and health status was unknown. Therefore, the purpose of this study was to explore the impacts of reimbursement policy expansion on hospice care use, and to investigate the effects on people with various demographics characteristics and health status.

**Methods:**

We used the 2001–2017 Taiwan NHI claims data, Death Registry, and Cancer Registry in this study, and we included people who died between 2002 and 2017. The study period was divided into 4 sub-periods. hospice care use and the initiation time of 1st hospice care use were used as dependent variables; demographic characteristics and health status were also collected.

**Results:**

There were 2,445,781 people who died in Taiwan during the study period. The results show that the trend of hospice care use increased over time, going steeply upward after the scope of benefits expansion, but the initiation time of 1st hospice care use did not increase after the scope of benefits expansion. The results also show that the effects of expansion varied among patients by demographic characteristics.

**Conclusion:**

The scope of benefits expansion might induce people’s needs in hospice care, but the effects varied by demographic characteristics. Understanding the reasons for the variations in all populations would be the next step for Taiwan’s health authorities.

## Introduction

Along with the aging of the population, the pattern of diseases that people suffer and die from is also changing. Increasingly, more people die as a result of serious chronic disease, and older people in particular are more likely to suffer from multi-organ failure toward the end of life, which causes a wide range of physical, psychological, and social problems [1]. Health care systems must be able to meet the needs of these people by reducing suffering and supporting people of all ages to live well and maintain their quality of life for as long as possible [1]. Aggressive treatment would create more burden than benefit for the patient at the end stage of life, and also would not be likely to improve survival. Hospice care, however, is designed to support the more personal aspects of this life stage: reflecting on one’s life and legacy, focusing on relationships in a deeper and more intentional way, achieving a sense of closure, and realizing any end-of-life goals. In this situation, the intervention of hospice care might be more likely to satisfy a patient’s needs and benefits, and dignity and quality of life could be improved.

Hospice care services have developed in many countries and have often been closely related to oncology. The worldwide need for this type of care remains much greater than the available provision of such care, but there are encouraging signs of recognition by policy makers and stakeholders, and interest in hospice care has never been greater [2]. Although the history of hospice care in Western countries is long and its use is well-developed, due to cultural differences, Taiwan has not had the same experience of a successful introduction of hospice care.

Hospice care services have been covered by Taiwan’s National Health Insurance (NHI) since 1996, and the Home-Based Hospice Care demonstration program was introduced first; later, in 2000, hospital-based service started to be reimbursed by NHI. Initially, NHI only covered hospice care for cancer patients; then amyotrophic lateral sclerosis and AIDS patients were enrolled. Since 2009, the NHI has expanded the scope of benefits to terminal patients with major organ failure. In addition, when the Hospice Palliative Care Act was passed by the Legislative Yuan on May 23, 2000, Taiwan became the first Asian country to pass such an act. The Taiwan Joint Commission on Hospital Accreditation has also added several items of hospice care in the accreditation protocol since the beginning of 2015 to require and encourage hospitals to provide high-quality hospice care. Lastly, the Patient Right to Autonomy Act took effect in January 2019, and Taiwan became the first country in Asia to pass a law promoting the concept of a “good quality of death.” Nowadays, hospice care services are provided for 10 categories of end-stage patients.

Although hospice care in Taiwan has been implemented for years, and many studies on it exist as well, most of the studies have focused on cancer patients as the study population, [3–5] exploring the trend of hospice care utilization [5–7] and its influence on medical costs [8–10]. Those studies usually used data from before the early 2010s, how did the influence of the scope of benefits expansion among various demographic groups and different causes of death was unknown; in addition, with an aging society and the advocacy of the government and NGOs, the attitude of society in Taiwan toward death has changed rapidly in recent years, and it might be necessary to reexamine the findings of previous studies. Therefore, the purposes of this study are to depict the trend of hospice care utilization from a long-term perspective, and to compare the utilization trend among various demographic characteristics and health status.

## Methods

### Study design

A retrospective, longitudinal population-level study design was conducted to fulfill the purposes of our study. We also divided the study period into 4 sub-periods: 2002–2005, 2006–2009, 2010–2013, 2014–2017.

### Data source and study population

We used the 2001–2017 NHI claims data, Taiwan’s Death Registry, and the Taiwan Cancer Registry in this study. The Death Registry was used to identified the study population; the Taiwan Cancer Registry was used to identify if a deceased person had had cancer or not; and the NHI claims data were used to identify the utilization of hospice care in the previous 12 months before a patient’s death.

People who died between 2002 and 2017 were included in this study. We identified their death events and the causes of death by consulting the National Death Registry database first, and we accessed the NHI claims data and the Taiwan Cancer Registry to retrieve the patients’ medical records. We used NHI claims data to identify whether a patient had received hospice care in the previous 12 months before that patient’s death, and we also used that data to retrieve each patient’s demographic characteristics. In addition, we used Taiwan Cancer Registry to retrieve a patient’s cancer-related information.

### Exclusion criteria

Deceased people who were under 18 years old, and deceased people for whom the information on gender, marital status and poverty status was unknown were excluded in this study.

**Definition of variables of interest**.

- Dependent variables.

There are 2 dependent variables used in this study: hospice care use, and the Initiation time of 1st hospice care use. The definitions of them are as follows:

#### Hospice care use

Hospice care use was defined as receiving any types of hospice care at least once in the last 12 months before death.

#### Initiation time of 1st hospice care use

The duration between 1st hospice care use and the date of death in the last year was used for calculation the initiation time.

b. Variables for stratified analysis.

We used demographic characteristics and health status for stratified analysis. Demographic characteristics include age of death, gender, marital status, poverty status, and urbanization of residence. Health status includes: history of having severe disease, and having the experience in receiving CPR in the last year. The details are following:

#### Age of death

Age of death was classified as *young adult* (18 to 40 years old), *middle-aged* (41 to 65 years old), *old* (66 to 85 years old), and *oldest old* (over 85 years old).

#### Marital status

Marital status was retrieved from the death registry, and there are 4 types of marital status: single, married, divorced, and widowed.

#### Poverty status

As for poverty, the registry for beneficiaries were used to distinguish patients in the low-income group from other populations. In Taiwan, the NHI Scheme classifies the insured into six insured categories according to the insured’s occupation. Households below the poverty line belong to classification 5. We used this information in the National Health Insurance Research Database as a criterion to identify the poverty status.

#### Urbanization of residence

A person’s residential area was linked to the urbanization level. Following Chang et al., [11] the actual location of each person is assumed in this study to be where an individual has the most outpatient and pharmacy visits. The location of each clinic and pharmacy is recognized as either urban or rural according to the definition of urbanization published by Taiwan’s National Health Research Institutes. All 365 townships in Taiwan are classified into 7 clusters based on the following indicators: population density (people/km^2^), proportion of people with a college degree or above, proportion of elderly people over 65 years of age, proportion of people who are agricultural workers, and the number of physicians per 100,000 people [12]. Residential areas located in clusters 1 to 3 were categorized as urban, others as rural.

#### History of having severe disease

Taiwan NHI’s catastrophic illness and rare disease certification, and cancer registry were used for identifying the history of having severe disease. If a person who can be identified in any of these two lists, then he/she was classified as having the history of severe disease.

#### Receiving CPR within a year before death

If a person who had ever received CPR within a year before his/her last month of life, then he/she was classified as receiving CPR within a year before death.

### Statistical analysis

In statistical testing, two-sided *P*-value ≤ 0.05 was considered statistically significant. The distributional properties of continuous variables were expressed by mean plus or minus standard deviation (SD), whereas categorical variables were presented by frequency and percentage. A chi-square test and two-sample *t*-tests were conducted to test the differences in hospice care utilization with respect to various people’s demographic characteristics and health status. All statistical analyses were performed using SAS (version 9.4, SAS Institution Inc., Cary, NC, USA).

## Results

Table 1 delineates the characteristics of the deceased population in this study. There were 2,289,965 people in Taiwan who died during the study period, with most of the deceased middle-aged or older (95%), male (61%), around third-fourth people were lived in urban areas, 63,274(2.76%) were identified as low-income, more than 80% of study population were married or widowed, more than 50% of them had severe diseases, and there were 17,719 (0.77%) had ever received CPR within a year before their last month. Finally, 253,658 (11.08%) received hospice care. Our data also show the average initiation time of 1st hospice care was 1.08 months. The distribution also shows that more than 60% of them had their first hospice care in in the same month of their death, the second was the one month before death, and the third was the second month before death, and so on. In the other word, the most majority of them received their first hospice care within three months before their death.

**Table 1:** Sample Description

Variable	statistics
Period, n (%)	
2002–2005	503,522(21.99)
2006–2009	540,863(23.62)
2010–2013	583,918(25.50)
2014–2017	661,662(28.89)
Age, n (%)	
18–40	113,527(4.96)
41–65	589,584(25.75)
66–85	1,100,780(48.07)
Above 86	486,074(21.23)
Gender, n (%)	
Male	1,394,485(60.90)
Female	895,480(39.10)
Urbanization level of residence, n (%)	
Urban	1,755,678(76.67)
Rural	534,287(23.33)
Poverty, n (%)	
Yes	63,274(2.76)
No	2,226,691(97.24)
Marital status, n (%)	
Single	220,194(9.62)
Married	1,237,114(54.02)
Divorced	146,598(6.40)
Widowed	686,059(29.96)
Severe disease, n (%)	
Yes	1,179,400(51.50)
No	1,110,565(48.50)
CPR history within 1 year, n (%)	
Yes	17,719(0.77)
No	2,272,246(99.23)
Hospice care use, n (%)	
No	2,036,307(88.92)
Yes	253,658(11.08)
Initiation time of hospice care, mean (S.D)	1.08(2.15)
Same month	156,331(61.63)
1 month before death, n (%)	44,284(17.46)
2 months before death, n (%)	18,710(7.38)
3 months before death, n (%)	10,237(4.04)
4 months before death, n (%)	6,327(2.49)
5 months before death, n (%)	4,306(1.70)
6 months before death, n (%)	3,183(1.25)
7 months before death, n (%)	2,354(0.93)
8 months before death, n (%)	1,869(0.74)
9 months before death, n (%)	1,389(0.55)
10 months before death, n (%)	1,263(0.50)
11 months before death, n (%)	1,113(0.44)
12 months before death, n (%)	2,292(0.90)

**Fig. 1:**
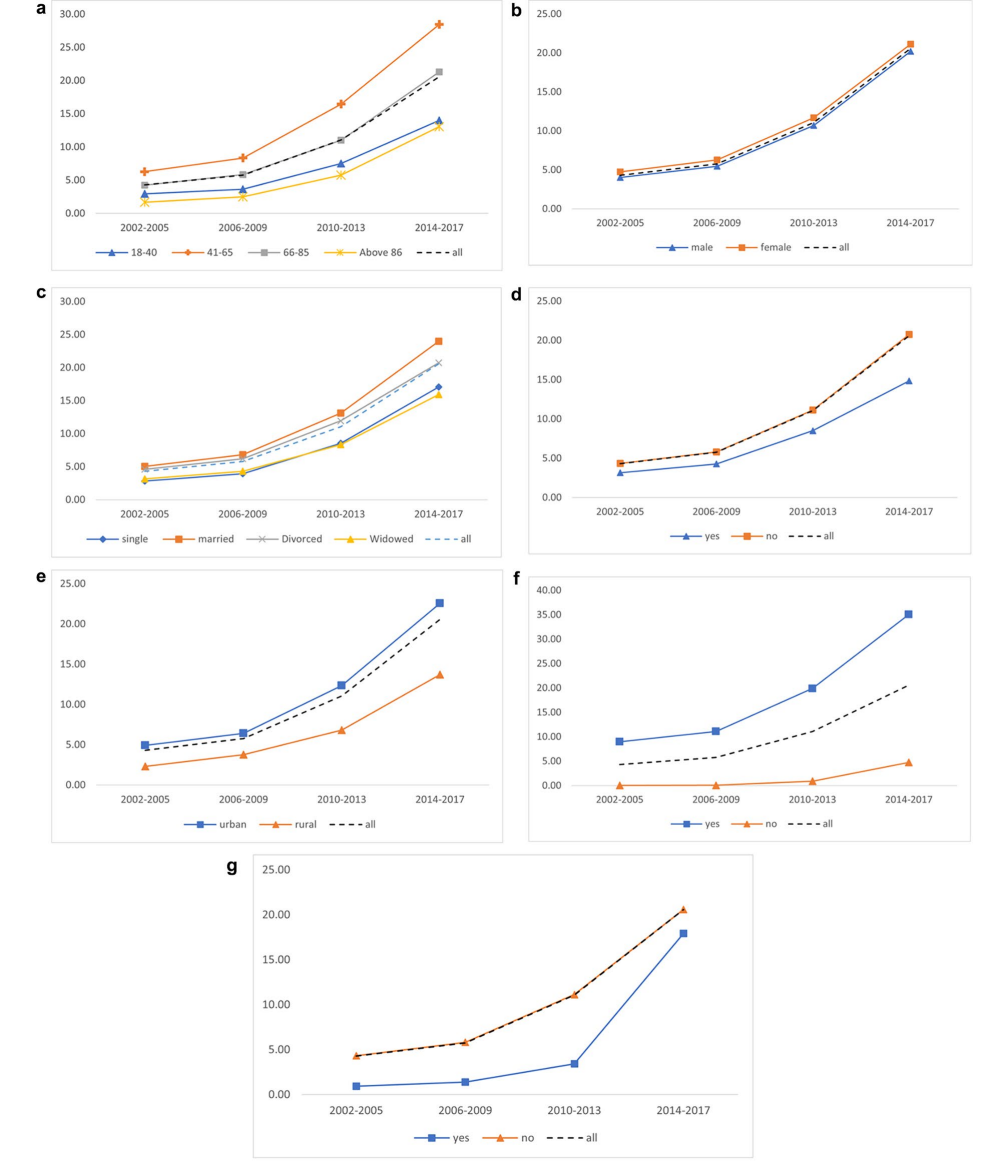
The Trend of the hospice care Use: by demographic characteristics and health status. a. The Trend of the hospice care Use: by age of death. b. The Trend of the hospice care Use: by gender. c. The Trend of the hospice care Use: by marital status. d. The Trend of the hospice care Use: by poverty status. e. The Trend of the hospice care Use: by urbanization of residence. f. The Trend of the hospice care Use: by history of severe disease. g. The Trend of the hospice care Use: by experience in receiving CPR

**Fig. 2:**
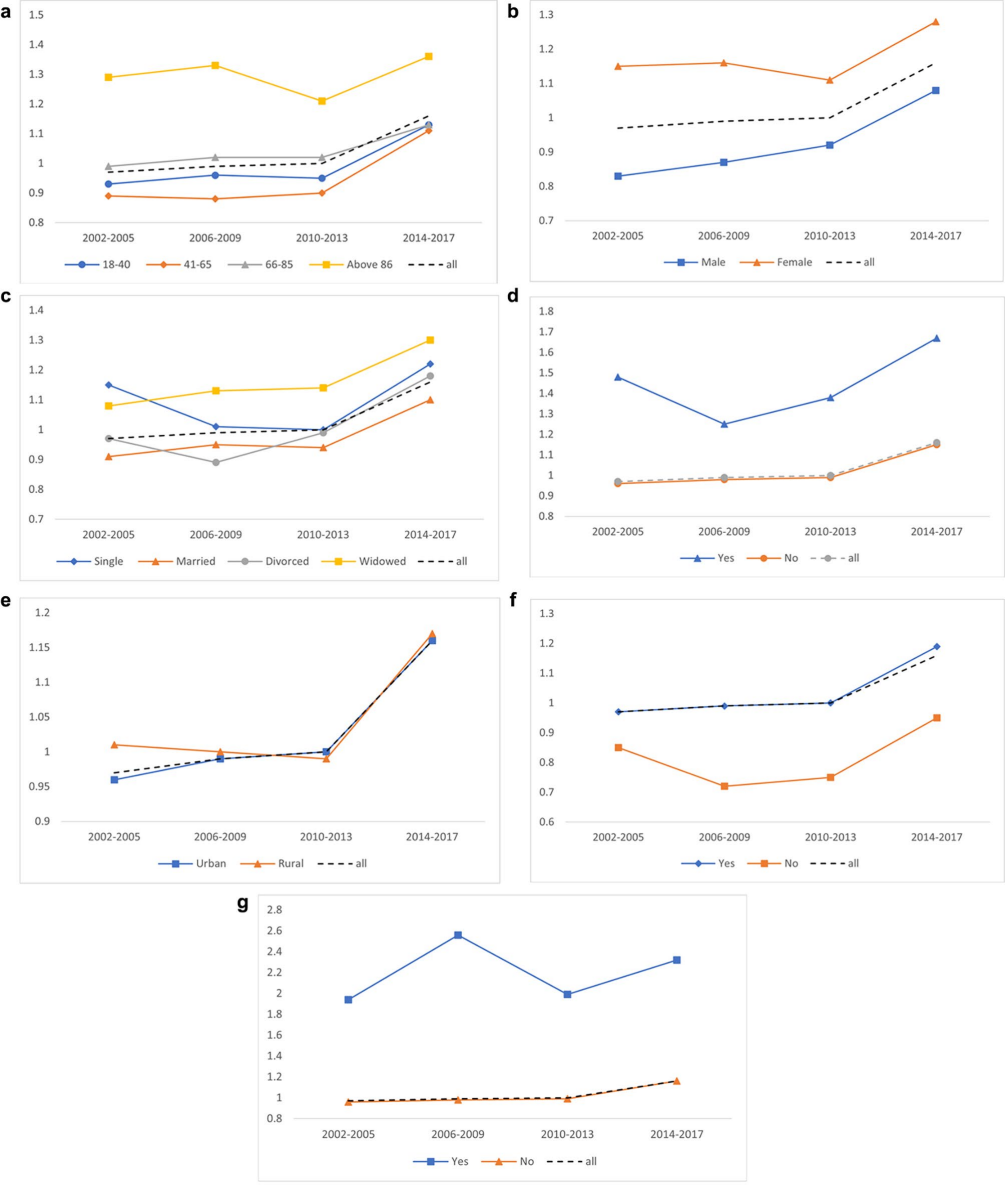
Initiation time of the 1st hospice care use: by demographic characteristics and health status. a. Initiation time of the 1st hospice care use: by age of death. b. Initiation time of the 1st hospice care use: by gender. c. Initiation time of the 1st hospice care use: by marital status. d. Initiation time of the 1st hospice care use: by poverty status. e. Initiation time of the 1st hospice care use: by urbanization of residence. f. Initiation time of the 1st hospice care use: by history of severe disease. g. Initiation time of the 1st hospice care use: by experience in receiving CPR.

Figure 1 demonstrates the trend of hospice care use among various demographic characteristics and health status. In general, we found the trend of hospice care use was increasing over time, and the growth rate was faster and faster among time periods. We also found the trend of hospice care use among various demographic characteristics and health status; some were more like to have hospice care and some were not. However, it is worth noting that the growth rate of various demographic characteristics and health status was varied as well. For example, people who were aged between 41 and 65, who were married, who had severe diseases, and who ever had received CPR, their growth rate of hospice care use were faster than average, people who were widowed, who were poor, rural dwellers, and who did not have cancer and other catastrophic illness and rare diseases were slower than average. The trend of the initiation time of the first hospice care use is presented in Fig. 2. The results show the initiation time was getting earlier over time. Likewise, the initiation time of hospice care was varied various demographic characteristics and health status, however, unlike the growth rates of hospice care use, the growth rates of the initiation time of hospice care were almost similar among various demographic characteristics and health status. (Fig. 2)

Table 2 shows the results of multivariate analysis of hospice care use. In general, our data show that as time went by, the use of hospice care was increased. Especially in the period of 2014–2017, the odds ratios increased dramatically (OR:6.59, 95% C.I.: 6.48–6.69), the effects of age on hospice care use seem reverse after the age of 65, the value of OR becomes smaller, from 1.48 (aged at 41–65 ) to 1.09 (aged at 66–85), and in the age group of above 86, the value of OR becomes 0.84 (95% C.I.: 0.81–0.87), which meant people who died at the age above 86 had less opportunity to utilize hospice care, after adjusting other demographic characteristics and health status. The results also reveal poor, and having the experience in receiving CPR in their last year were negatively associated with hospice care use, have/had marital relationship, and have the history of severe disease were positively associated with hospice care use. We also stratified our data by periods, the results of stratified analysis were also similar with overall data. The results also reveal that the impacts of the scope of benefits expansion on the use of hospice care varied among patients of different demographic characteristics and health status groups. Some are getting closer (e.g., having the history of severe disease, having the experience in receiving CPR in the last year) and some are getting wider (e.g., poverty status, urbanization of residence), and some are mixed.

**Table 2:** Results of Multivariate Analysis with stepwise selection: hospice care use

	Overall	2002–2005	2006–2009	2010–2013	2014–2017
Period (ref = 2002–2005)					
2006–2009	1.30 (1.28,1.33)				
2010–2013	2.73 (2.68,2.77)				
2014–2017	6.59 (6.48,6.69)				
Age (ref = 18–40)					
41–65	1.48 (1.44,1.53)	1.30 (1.21,1.40)	1.38 (1.29,1.48)	1.48 (1.40,1.56)	1.58 (1.51,1.65)
66–85	1.09 (1.06,1.12)	1.05 (0.98,1.13)	1.09 (1.01,1.16)	1.04 (0.98,1.10)	1.14 (1.09,1.19)
Above 86	0.84 (0.81,0.87)	0.62 (0.57,0.68)	0.66 (0.61,0.72)	0.75 (0.71,0.80)	0.95 (0.91,0.99)
Gender (ref = male)	1.18 (1.17,1.19)	1.22 (1.19,1.26)	1.22 (1.19,1.25)	1.18 (1.16,1.21)	1.15 (1.13,1.17)
Urbanity (ref = rural)	1.60 (1.58,1.62)	1.64 (1.58,1.71)	1.39 (1.35,1.44)	1.61 (1.58,1.65)	1.62 (1.59,1.65)
Poverty (ref = no)	0.66 (0.64,0.68)	0.83 (0.75,0.93)	0.75 (0.69,0.82)	0.70 (0.66,0.75)	0.61 (0.58,0.63)
Marital status (ref = single)					
Married	1.27 (1.25,1.30)	1.21 (1.14,1.29)	1.26 (1.20,1.33)	1.27 (1.23,1.32)	1.27 (1.24.1.31)
Divorced	1.15 (1.12,1.17)	1.35 (1.24,1.47)	1.32 (1.23,1.41)	1.19 (1.13,1.24)	1.07 (1.04,1.11)
Widowed	1.08 (1.06,1.11)	1.10 (1.03,1.18)	1.11 (1.05,1.18)	1.11 (1.07,1.16)	1.05 (1.02,1.08)
Severe disease (ref = no)	15.07 (14.83,15.31)	571.67 (420.82,776.58)	204.52 (173.62,240.92)	24.47 (23.49,25.49)	9.85 (9.68,10.03)
CPR (ref = no)	0.28 (0.26,0.30)	0.12 (0.09,0.16)	0.14 (0.11,0.18)	0.17 (0.14,0.20)	0.49 (0.45,0.54)

Odds ratio (95% confidence interval); ref = reference

As for the initiation time of hospice care use, our data reveal that age of death, gender, poverty status, having the history of severe disease, have the experience in receiving CPR were positively associated with the initiation time of 1st hospice care use, the status of married was negatively associated with the initiation time of 1st hospice care use. And we also found the effects of demographic characteristics and health status on the initiation time of 1st hospice care use were almost consistent, except for the status of poverty and having the experience in receiving CPR in the last year. The results of stratified analysis were also similar with overall data, except for the effects of marital status became insignificant. (See Table 3)

**Table 3:** Results of Multivariate Analysis with stepwise selection: initiation time of hospice care

	Overall			2002–2005			2006–2009			2010–2013			2014–2017	
Period (ref = 2002–2005)														
2006–2009	0.01(0.02)													
2010–2013	0.02(0.02													
2014–2017	0.20(0.02)	^***^												
Age (ref = 18–40)														
41–65	< 0.01(0.03)		-0.02(0.07)			-0.07(0.06)			-0.04(0.05)			-0.02(0.04)		
66–85	0.09(0.03)	^**^		0.09(0.07)			0.07(0.06)			0.09(0.05)			0.03(0.04)	
Above 86	0.36(0.03)	^***^		0.37(0.08)	^***^		0.37(0.07)	^***^		0.32(0.05)	^***^		0.34(0.04)	^***^
Gender (ref = male)	0.21(0.01)	^***^		0.33(0.03)	^***^		0.29(0.02)	^***^		0.20(0.02)	^***^		0.20(0.01)	^***^
Poverty (ref = no)	0.51(0.03)	^***^		0.54(0.10)	^***^		0.35(0.08)	^***^		0.48(0.05)	^***^		0.60(0.04)	^***^
Marital status (ref = single)														
Married	-0.08(0.02)	^***^												
Divorced	-0.01(0.02)													
Widowed	-0.03(0.02)													
Severe disease (ref = no)	0.39(0.02)	^***^								0.39(0.04)	^***^		0.38(0.02)	^***^
CPR (ref = no)	1.17(0.08)	^***^		0.99(0.28)	^**^		1.63(0.23)	^***^		1.00(0.17)	^***^		1.17(0.10)	^***^

Beta (standard error); *p < 0.05; **p < 0.01; ***p < 0.001; ref = reference

## Discussion

In our study, we used 16 years of data to depict the trend of hospice care use in Taiwan, and we observed the trends in hospice care use among patients with different demographic characteristics. The results of this study show that the trend of hospice care increased over time, especially after the scope of benefits expansion in 2009, and that the growth rate after 2010 was significantly greater than it was between 2002 and 2010. In addition, people who were between the ages of 40 and 65, female, married or divorced, and not poor were more likely to receive hospice care. On the other hand, the oldest old, those who did not die from cancer, and non-urban dwellers were less likely to make use of such care. Furthermore, the impacts of the scope of benefits expansion on the duration of hospice care use among various population characteristics varied. These findings could offer evidence that could aid policy makers in figuring out how to improve the use of hospice care in Taiwan.

We also found that the utilization rate of hospice care suddenly increased after 2010. It could be concluded that the increase happened as a result of Taiwan’s NHI expanding the scope of benefits to terminal patients with major organ failure. However, it cannot be ignored that the need for hospice care still exists in Taiwanese society. Therefore, when the the scope of benefits for hospice care was expanded, the increasing utilization rate was expected. Further, we also found that people who were between 40 and 65 years old were more likely to receive hospice care. That younger people were less likely to receive hospice care is understandable, as people may exhaust all possible options to save a young person’s life [13]. But why were patients over the age of 85 not prone to using hospice care? This finding is contrary to previous studies [14–16]. This phenomenon might be explained from a sociocultural perspective [17, 18]. Filial piety, or Chinese parental respect, is a core value in Taiwan society. Children are expected to be nice to their parents, or to obey their parents. Even though hospice care has been gradually accepted in Taiwanese society, some people still conflate hospice care with either euthanasia or assisted suicide. Because of this, elderly relatives may oppose the use of hospice care, and the children opting for hospice care may not be considered good sons or daughters.

Regarding how marital status fits into the use of hospice care, the findings of previous studies were not consistent: Some studies demonstrated that married people tended to use hospice care service more than single people, [19–21] but some studies found there was no association between marital status and use of hospice care [22, 23]. Our results showed that married people tended to use hospice care more than those who were single, existed studies have found people with stronger support might be more likely to choose hospice care, [24] whether married people have better family support is an issue worth to discuss. Last, our results also revealed that people who had cancer or had a poor health status were prone to using hospice care, which was consistent with existing studies [25–27].

Poverty is a significant barrier to seeking healthcare in general, and hospice care is no exception. Some studies have found that poor people underutilize hospice care [28–30]. A person’s place of residence is another issue to be considered in relation to access to healthcare [31–34]. Healthcare resources are usually concentrated in urban areas; therefore, urban dwellers usually find that hospice care is more available to them than to non-urban dweller, and the closer proximity to places that offer hospice care might explain that difference. Income status and residential area are quite often used to measure the level of socioeconomic status. Previous studies have shown that a lower socioeconomic status was associated with poor health literacy, [35] and some studies on hospice care have found that better health literacy comes along with better health utilization, [36] which could support our findings.

The timing of hospice care initiation is an issue worth discussing. Several studies we reviewed suggested that hospice care should be initiated in enough time before death [37–39]. But our findings revealed the average duration of hospice care use was only around 1 to 2 months, which might imply that use of hospice care in Taiwan has a long way to go. Although the idea of hospice care in Taiwan has been introduced over decades, it has only been gradually accepted by Taiwanese society in the past ten years. The concept of hospice care was often misunderstood as giving up treatment, and even now, this phenomenon still exists. As we mentioned above, elderly relatives may oppose the use of hospice care, some children are also under tremendous social pressure when making decisions for their parents. Therefore, the timing of hospice care intervention is quite late, even though hospice care has been gradually accepted by more and more Taiwanese people, their understanding of hospice care is still not very accurate. Helping people have a better understanding of hospice care could require significant time and effort.

### Limitations

Although our study used long-term data to observe the trend and duration of hospice care use among various demographic groups, and found out that the scope of benefits expansion in 2009 did influence hospice care use, the effects were inconsistent among different demographic groups. But this study also faced some limitations. First of all, there were some important factors that were not observable, such as cultural and religious factors, and family support, which were commonly mentioned in the literature, [40] but were not collectable from claims data. The lack of this information may affect the results of this study. Secondly, there is the limitation of secondary data. The major strengths of secondary data include lower cost, larger sample sizes, and easier follow-up over time. However, there are also several limitations and challenges in dealing with claims data, such as the gap between the actual situation and the database, which might affect the validity of our findings. Last, the data analyzed is exclusive of sexual orientation and gender identity data. This indicates that research investigating the influencing factors of hospice care utilization for people who are sexual and gender minority is needed.

## Conclusion

In this study, we found that the utilization of hospice care in Taiwan increased over time. We also found that the scope of benefits expansion in 2009 did influence the utilization of hospice care; however, the effects of the scope of benefits expansion varied. This depends on population demographic characteristics and health status. Conservative cultural background, misunderstanding of hospice care, and unequal distribution of hospice care facilities might have influences on the attitude and accessibility of hospice care for people with some certain characteristics. Health authorities should propose a set of strategies for eliminating the differences in the utilization of hospice care to avoid the disparity issue occurred in the future.

## Data Availability

The data that support the findings of this study are available from Ministry of Health Welfare Taiwan but restrictions apply to the availability of these data, which were used under license for the current study, and so are not publicly available. Data are however available from the authors upon reasonable request and with permission of the Ministry of Health Welfare Taiwan. (https://dep.mohw.gov.tw/dos/cp-2516-59203-113.html)
